# Urban landscapes increase dispersal, gene flow, and pathogen transmission potential in banded mongoose (*Mungos mungo*) in northern Botswana

**DOI:** 10.1002/ece3.7487

**Published:** 2021-06-29

**Authors:** Kelton Verble, Eric M. Hallerman, Kathleen A. Alexander

**Affiliations:** ^1^ Department of Fish and Wildlife Conservation Virginia Tech Blacksburg VA USA; ^2^ Chobe Research Institute CARACAL Kasane Botswana; ^3^ Present address: Department of Biological Sciences University of Alabama Tuscaloosa AL USA

**Keywords:** behavior, Chobe, dispersal, microsatellite DNA, *Mycobacterium mungi*, noninvasive sampling, population structure, urban landscapes

## Abstract

Disease transmission can be strongly influenced by the manner in which conspecifics are connected across a landscape and the effects of land use upon these dynamics. In northern Botswana, the territorial and group‐living banded mongoose (*Mungos mungo*) lives across urban and natural landscapes and is infected with a novel *Mycobacterium tuberculosis* complex pathogen, *M. mungi*. Using microsatellite markers amplified from DNA derived from banded mongoose fecal and tissue samples (*n* = 168), we evaluated population genetic structure, individual dispersal, and gene flow for 12 troops. Genetic structure was detectable and moderately strong across groups (*F*_ST_ = 0.086), with *K* = 7 being the best‐supported number of genetic clusters. Indications of admixture in certain troops suggest formation of new groups through recent fusion events. Differentiation was higher for troops inhabiting natural areas (*F*_ST_ = 0.102) than for troops in urban landscapes (*F*_ST_ = 0.081). While this suggests increased levels of gene flow between urban‐dwelling troops, the inclusion of a smaller number of study troops from natural land types may have influenced these findings. Of those individuals confirmed infected with *M. mungi*, the majority (73%, *n* = 11) were assigned to their natal group which is consistent with previous observations linking lower levels of dispersal with infection. Twenty‐one probable dispersing individuals were identified, with all suspected migrants originating from troops within the urban landscape. Findings suggest that urbanized landscapes may increase gene flow and dispersal behavior with a concomitant increase in the risk of pathogen spread. As urban landscapes expand, there is an increasing need to understand how land use and pathogen infection may change wildlife behavior and disease transmission potential.

## Graphical abstract

Using microsatellite markers amplified from DNA derived from banded mongoose fecal and tissue samples (*n* = 168), we evaluate gene flow in banded mongoose (*Mungos mungo*) in northern Botswana. This species lives across urban and natural landscapes and is infected with a novel Mycobacterium tuberculosis complex pathogen, *M. mungi*. Our results suggest that urban landscapes may increase mongoose movements between groups, gene flow, and potentially pathogen transmission potentially highlighting the critical influence urban landscapes may have on wildlife behavior and ecology.

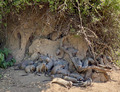

## 1 INTRODUCTION

Our ability to predict infectious disease dynamics remains limited, particularly in free‐ranging wildlife populations. Pathogen transmission processes can be complex, variable, and strongly shaped by attributes of interactions among hosts, pathogens, and their environments. Across host species, sociality can have an important influence on pathogen transmission dynamics, determining the degree of connectivity within and between groups, which in turn can influence the potential for pathogen spread (Cremer et al., 2007; Galvani, 2003; Loehle, 1995; Reiner et al., 2013; Sanderson et al., 2014; Sattenspiel & Simon, 1988). A central feature shaping these connections is the manner in which individuals or groups of individuals disperse across a landscape, moving from their natal area to establish themselves in another area or habitat patch. Here, landscape type and structure can interact with species behavior, shaping these movements and connections within and between groups, modifying disease transmission potential, and epidemic dynamics. The influence of land type can be significant, as for example in human‐modified landscapes where the dispersal potential of a species may be either hindered under certain circumstances (Forman et al., 2003; Hamer & McDonnell, 2008) or increased in others (McKinney, 2006). Natural landscape features also may limit animal movement and consequently transmission and spread of pathogens. For example, spread of rabies in raccoon (*Procyon lotor)* populations was inhibited by large river courses that blocked raccoon movements (Côté et al., 2012, Hirsch et al., 2013; Rioux Paquette et al., 2014). Spatial and temporal landscape variation may significantly influence a species movement behavior across their range, limiting the accuracy of regional dispersal estimates and model‐based predictions of pathogen spread (Bowler & Benton, 2005). The complexity of these host–pathogen–landscape interactions continues to challenge our ability to computationally characterize dispersal in predictive infectious disease models.

**FIGURE 1:**
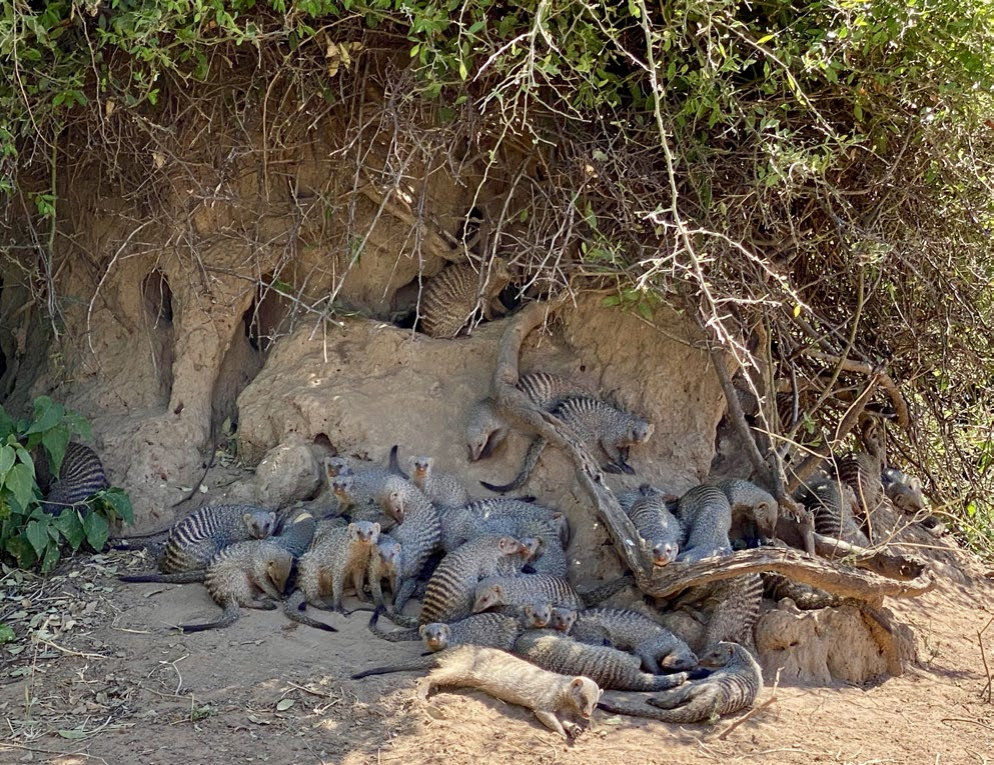
Banded mongooses are small, territorial carnivores that lives in social groups

The banded mongoose, *Mungos mungo* (Figure 1), is a small, fossorial carnivore that lives in social groups that can number from 5 to 75 individuals (Laver & Alexander, 2018). This territorial species has a predominantly egalitarian social system, with low reproductive skew (Cant, 2000). In northern Botswana, banded mongooses are infected with a novel tuberculosis pathogen *Mycobacterium mungi,* a member of the *M. tuberculosis* complex (Alexander et al., 2016). This tuberculosis (TB) pathogen is transmitted primarily through infected scent marks and associated contact that arises from olfactory communication behaviors. This population lives across a mixed land use area including both natural ecosystems and urbanized areas, with movement behavior of mongooses varying according to land type and proximity to humans (Laver & Alexander, 2018). Evidence from previous studies (Fairbanks, 2013) suggests that mongoose dispersal behavior is increased in this region, differing significantly from that of populations living in a protected area in Uganda, where mongoose dispersal is extremely limited (Gusset, 2007). Occurrence of TB disease in the Botswana systems appears also to affect dispersal behaviors, with clinically ill mongooses dispersing less frequently than healthy mongooses (Fairbanks et al., 2014). While more data are needed, there was also evidence to suggest that healthy individuals residing in troops with more infected mongooses may be more likely to disperse than individuals in troops with lower infection levels (Fairbanks et al., 2014). Bidirectional interactions between dispersal and disease may have critical implications for infectious disease dynamics, information central to understanding and predicting epidemic dynamics.

Given the apparent complexity of mongoose movement behaviors in this system, we used microsatellite DNA markers as a tool to characterize troop and local‐scale population structure and to infer patterns of dispersal and genetically effective migration among troops. We predicted a priori that (1) human‐dominated landscapes would increase mongoose dispersal above that observed in natural landscapes, and (2) the occurrence of infectious disease would have a negative association with dispersal behaviors in infected mongooses, with sick mongooses being assigned to their putative natal troop. Our aim was to use this model system to evaluate the influence of landscape and infection on dispersal dynamics and our understanding of pathogen transmission and persistence dynamics in transforming landscapes.

## 2 MATERIALS AND METHODS

### 2.1 Study area and species

**FIGURE 2:**
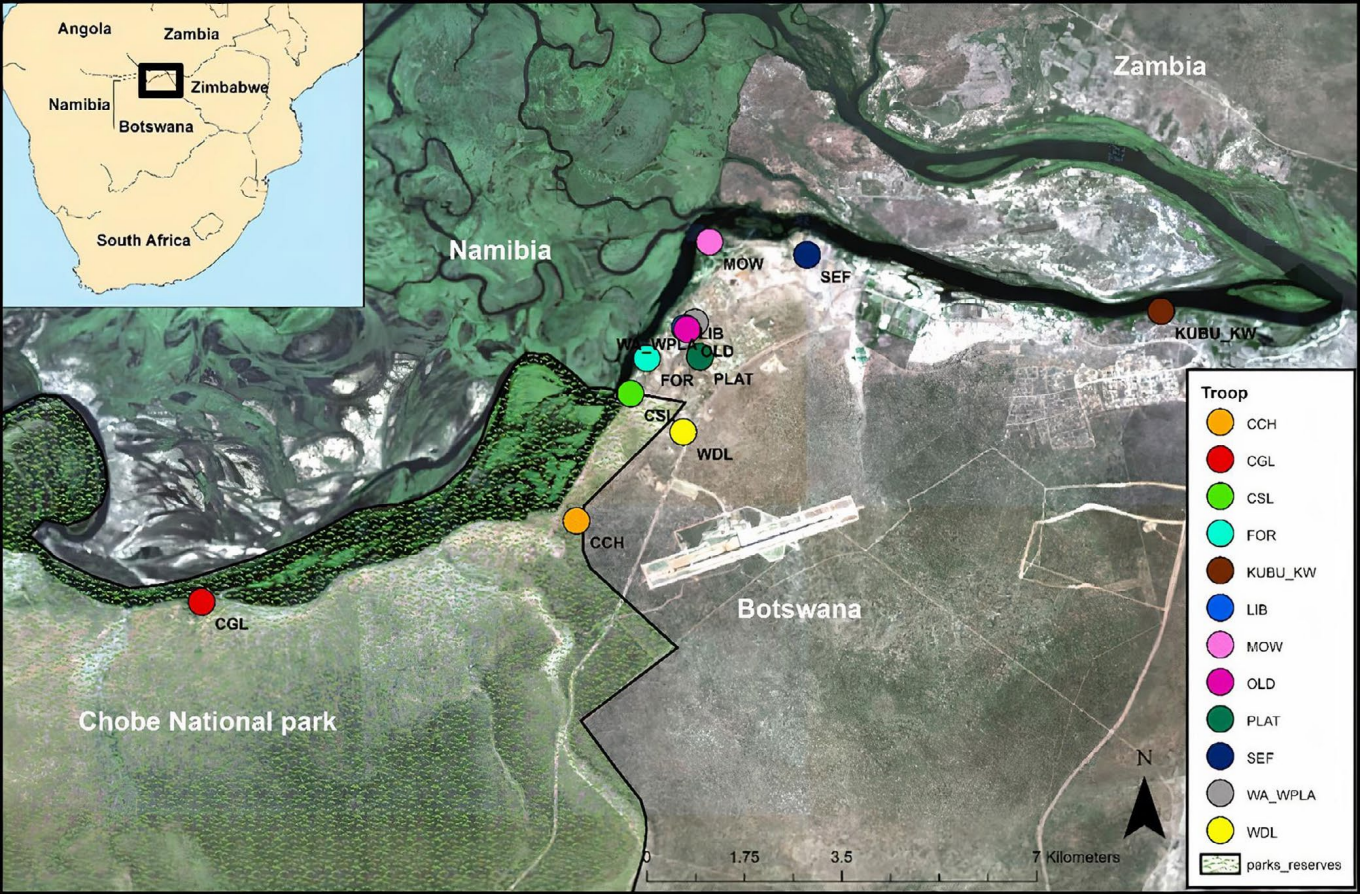
Spatial distribution of sampled lartrine sites (colored circles) belonging to 12 troops of mongooses (at time of fecal sample collection) along the Chobe River in northern Botswana

Our long‐term study site is located in the northern part of Botswana in Chobe District (Figure 2). Mongoose troops in our study area occur across protected and unprotected landscapes, including the growing urban center of Kasane. Since the discovery of *M. mungi* in 2000, troops in the region have been persistently infected with this pathogen, making them the focus of long‐term studies (Alexander et al., 2010; Laver & Alexander, 2018).

### 2.2 Sample collection

Mongoose troops were monitored weekly through the use of VHF radio collars that were fitted to one or two individuals in each study troop as previously described (Laver & Alexander, 2018). Banded mongooses use latrine sites for defecation, providing a localized area to collect fecal samples from a majority of troop members in one event (Pesapane et al., 2013). The evening prior to a sampling event, a troop of interest was selected and tracked to their denning site using radio telemetry. Existing fecal boluses that could be detected were removed from the latrine site so that fresh samples could be collected the following morning with reduced risk of cross‐contamination. To obtain samples that were fresh and with the least host DNA degradation, troops were observed as they emerged from the den shortly after sunrise. Mongooses that exited the den were counted and monitored to see where they moved and to observe signs of defecation (squatting, lifted tail, etc.). The latrine site was approached for sample collection at least 10 min after the last individual left to ensure that most members had the opportunity to defecate without disturbance. Even after leaving the communal latrine site, the troop of interest was followed in case individuals defecated in another location. Distinguishing fresh fecal samples from older samples was based on color, firmness, and moisture of the stool (Pesapane et al., 2013). Using a sterilized surgical blade, the outer surface of each fecal bolus was carefully removed, avoiding surfaces in contact with the soil, which can act as a PCR inhibitor (Pesapane et al., 2013). The fecal matter was transferred into a sterilized 1.5‐ml microcentrifuge tube and placed in a portable cooler with ice packs.

Blood and tissue samples were also obtained from banded mongooses sampled during capture activities, opportunistic postmortems (individuals subject to hit by car, dog attack, or human persecution), or collected in association with other management activities. Each sample was assigned an animal identification number, and data were recorded regarding sex of the animal, age class (juvenile, sub‐adult, adult), date of capture, and affiliated troop at the time of capture or carcass discovery. *M. mungi* infection status can only be determined postmortem, with infection status assigned based on the presence of clinical disease, postmortem TB lesions, and/or the detection of *M. mungi* DNA in tissues and/or secretions as previously described (Alexander et al., 2016). Individuals genotyped by fecal samples were, therefore, not given an infection status. Similarly, while we can identify sick animals based on the above criteria, we cannot definitively identify animals as being free from infection. We, therefore, restricted our examination of dispersal and disease to those mongooses that had died and had their infection status specifically determined.

Fecal DNA was extracted using the PowerSoil DNA Isolation Kit (MoBio^®^). DNA samples were stored in a −20°C freezer. Extractions were done within 24–48 hr in an attempt to obtain the highest quality and yield of host DNA from each sample. For DNA originating from blood or tissue, Quick‐DNA Plus Kits (Zymo^®^) were used for the extraction process. Both kits were used according to the manufacturer's specifications.

### 2.3 Genotyping

Genotyping was performed at 20 microsatellite loci (Griffin et al., 2001, Sanderson et al., 2015; Waldick et al., 2003; Table 1) derived from previous studies of banded mongooses and other members of Family Herpestidae. Individual samples were genotyped in three multiplexed polymerase chain reactions (PCR). To ensure strong amplification at each locus without allelic dropout or other molecular artifacts, amplification conditions for each multiplex were optimized by assessing the fluorescence of amplicons subjected to electrophoresis in an ethidium bromide‐stained 3.5% agarose gel with a 100 base pair molecular weight ladder. Each reaction was carried out in a 10‐µl reaction consisting of 5 µl of Qiagen Multiplex PCR Kit buffer, 1 µl of a 10× primer mix, 1 µl of bovine serum albumin (BSA), 2 µl of water, and 1 µl of 5ng DNA template. The conditions for the PCR protocol were as follows: 1 cycle of initial activation for 15 min at 95°C, followed by 34 cycles of denaturation for 30 s at 95°C, annealing for 45 s at 57°C, and extension for 30 s at 72°C, and a final extension for 10 min at 72°C. Forward primers were fluorescently labeled with 6‐FAM, NED, PET, or VIC dyes, ensuring that different primers with similar product lengths (within 10 base pairs) would exhibit different labeling. The PCR products were sent to the Cornell University Biotechnology Resource Center for amplification fragment size analysis using an ABI3730 Genetic Analyzer.

**TABLE 1:** Sources, genetic variability metrics across all troops within the study area, and results of analysis of molecular variance (AMOVA) for polymorphic loci used for genetic analysis of banded mongoose troops in the Chobe district of northern Botswana: average observed heterozygosities (*H_O_*) and expected heterozygosities (*H_E_*), average number of alleles (*A*), and average allelic size range. Results of partitioning of genetic variation using AMOVA are displayed as % variation among troops, among individuals, and within individuals

Locus	Species origin	Source	*H_O_*	*H_E_*	*A*	Range	% Variation		
							Among troops	Among individuals	Within individuals
*Mon‐16*	*Mungos mungo*	Sanderson et al. (2015)	0.584	0.618	3.67	6.17	11.2	5.3	83.5
*Mon‐19*	*Mungos mungo*	Sanderson et al. (2015)	0.753	0.715	5.00	13.33	10.7	−4.4	93.7
*Mon‐25*	*Mungos mungo*	Sanderson et al. (2015)	0.723	0.783	5.75	11.50	5.6	8.2	86.2
*Mon‐32*	*Mungos mungo*	Sanderson et al. (2015)	0.466	0.568	3.08	4.16	0.1	13.1	86.8
*Mon‐38*	*Mungos mungo*	Sanderson et al. (2015)	0.713	0.786	5.17	10.00	2.0	8.2	89.8
*Mon‐41*	*Mungos mungo*	Sanderson et al. (2015)	0.323	0.398	2.91	5.82	7.7	12.4	79.9
*Mon‐65*	*Mungos mungo*	Sanderson et al. (2015)	0.339	0.304	2.56	7.56	15.1	−11.6	96.5
*Mon‐66*	*Mungos mungo*	Sanderson et al. (2015)	0.759	0.690	4.55	12.91	11.5	−9.0	97.5
*Mon‐68*	*Mungos mungo*	Sanderson et al. (2015)	0.702	0.695	4.42	19.83	7.5	−0.3	92.8
*Mon‐69*	*Mungos mungo*	Sanderson et al. (2015)	0.539	0.484	3.17	28.67	14.3	−8.6	94.3
*Mon‐70*	*Mungos mungo*	Sanderson et al. (2015)	0.751	0.789	6.42	33.00	7.263	2.255	90.5
*Mm2‐10*	*Mungos mungo*	Waldick et al. (2003)	0.631	0.729	4.75	20.17	4.6	16.0	79.4
*Mm10‐7*	*Mungos mungo*	Waldick et al. (2003)	0.423	0.532	3.83	6.67	13.9	15.7	70.5
*Ss11‐12*	*Suricata suricatta*	Griffin et al. (2001)	0.580	0.525	4.00	57.09	7.6	−5.1	97.5
*Ss13‐8*	*Suricata suricatta*	Griffin et al. (2001)	0.657	0.791	6.92	24.17	9.9	16.1	74.0
						Mean	8.2	4.6	87.2

Given the small amounts of host DNA in fecal samples (Bellemain et al., 2005; Waits & Paetkau, 2005), a multiple‐tube amplification approach (Navidi et al., 1992; Tab erlet et al., 1996; Watts et al., 2011) was implemented to ensure accurate and repeatable host genotyping from mongoose fecal samples. From results of an initial PCR, if an individual was scored as heterozygous at a given locus, it was recorded as heterozygous. Results for loci that were scored as homozygous were compared to results of a second PCR for the same individual; if the genotype was heterozygous in the second PCR, then the individual was recorded as heterozygous at that locus. Individuals for whom both PCRs indicated homozygosity for the same allele at the same locus were considered homozygous. If an individual was homozygous at one allele for a locus for the initial PCR and homozygous for a different allele at the same locus for the additional PCR, the individual was recorded as heterozygous for the observed alleles at that locus. We interpreted results showing three or more alleles at one locus across the two PCR amplifications as having been cross‐contaminated with another sample, and those results were removed from subsequent analyses.

### 2.4 Genetic analysis

Each individual's genotyping file was manually uploaded, and amplicon sizes were visualized and scored using GeneMarker ver 2.6.2 (Holland & Parson, 2011); peaks were scored automatically using the default software settings and a GS‐500 size standard (Applied Biosystems). Fluorescence peaks were manually scored on three separate occasions to ensure consistent allele calling. Since fecal samples were collected without knowing the individual donor, we screened for any duplicate samples using Microsatellite Toolkit (Park, 2001). If fewer than four alleles differentiated individuals, then they were considered duplicates. Any duplicated multilocus genotypes were removed from the dataset.

MICROCHECKER (Van Oosterhout et al., 2004) was used to assess the possibility of genotyping errors attributed to null alleles, large allele dropout, and accidental scoring of stutter peaks and to estimate frequencies of any null alleles.

Exact tests of deviation from Hardy–Weinberg and linkage equilibria, and calculation of observed and expected heterozygosities were conducted using Arlequin ver. 3.5 (Excoffier & Lischer, 2010). Microsatellite Toolkit (Park, 2001) and Arlequin provided allelic size ranges, and Arlequin calculated Garza and Williamson (2001) *m* indices for each locus and troop. Private alleles—those occurring in only one troop—were identified using GeneAlEx 6.5 (Peakall & Smouse, 2012). Estimates of fixation indices, genetic distances between populations, and analysis of molecular variance (AMOVA) were conducted using Arlequin. We tested for the effects of isolation‐by‐distance by regressing both *F*_ST_ and *F*_ST_/(1 – *F*_ST_) on geographic distance between sampling locations (Diniz‐Filho et al., 2013).

STRUCTURE (Pritchard et al., 2000) was used to assess population structuring across the study area by assigning multilocus genotypes of individual mongooses to given numbers of subpopulations using Bayesian clustering approaches. Posterior support for various numbers of clusters (*K*) from *K* = 1 to 15 was evaluated, with support for each *K* tested using 10 iterations. Each iteration was tested using 50,000 burn‐ins and 500,000 Markov chain Monte Carlo (MCMC) repetitions. GeneClass2 (Piry et al., 2004) was used for similar and complementary purposes to estimate inferred population of individual origin and identification of apparent migrants. This program utilizes Bayesian and distance‐based criteria to assess demographic structure of populations and uses Monte Carlo resampling for probability computations. Both STRUCTURE and GeneClass2 were applied to detect migrant individuals within each population cluster. Program R (R Core Team, 2013) was used to conduct Fisher's exact test to test for significance between migrant individuals dispersing from town troops and those from troops living within the park.

Rather than solely relying on the genotypic data to create clusters of genetically similar individuals, the LOCIPRIOR model (Pritchard et al., 2003), which is within the admixture model of STRUCTURE, was also employed in identical runs. LOCIPRIOR uses prior information regarding the location, or troop, from which each sample was collected to better inform the clustering algorithm. With the LOCIPRIOR model, STRUCTURE takes into consideration that the sampling location may be the true location of origin for each individual, as long as genetic data also provide support for this claim. The LOCIPRIOR model is appropriate for populations where detectable structuring may be weak when relatively few individuals are sampled per group or location, which is important to consider given the limited scale of our study site.

Contemporary effective population sizes (*N*_e_) were estimated using NeEstimator ver. 2 (Do et al., 2014). We applied the single‐sample method of Waples and Do (2010) which uses a random linkage disequilibrium (LD) approach that has been shown to have high precision for microsatellite data.

GROUPRELATE (Valsecchi et al., 2002) was used to assess the relatedness of individuals within and across troops. This Excel macro algorithm performs 1,000 randomizations to establish average relatedness values by generating new genotypes from the presented allele frequency distributions. Within‐group relatedness was estimated for each troop as well as pairwise values of between‐troop relatedness. Values of *r* > 0.25 are indicative of potential first‐ and second‐degree relationships, while conversely, values of *r* < −0.25 suggest no relation. GROUPRELATE is particularly useful for identifying patterns of relatedness between and within sexes; however, sex assignment was not available for the majority of individuals because the respective DNA samples were obtained from fecal samples collected at communal latrine sites and could not be associated with particular individuals.

### 2.5 Study permissions

Methods for this study were approved by the Virginia Tech Institutional Care and Use Committee (#16‐217‐FIW). Research clearance was provided by the Botswana Ministry of Environment, Natural Resources Conservation, and Tourism (EWT 8/36/4 XXXVIII).

## 3 RESULTS

### 3.1 Genotyping and loci metrics

From the 167 individual fecal samples collected, 77% were successfully amplified at a minimum of 13 loci. DNA from 49 blood or tissue samples, each representing a unique individual with sex and age‐class data, was also genotyped and included in the dataset. After identification and removal of nine duplicate samples, multilocus data from 168 individuals across 12 troops were used in analyses.

Due to a lack of polymorphism or unreliable PCR amplification, data from five of the original 20 microsatellite loci (*MmAAC5, Mm18‐1, Mm7‐5, Mon‐67,* and *Mm19*) were omitted from subsequent analyses, leaving data for 15 loci (Table 1). MICROCHECKER detected the segregation of null alleles at loci *Mm10‐7* (*n* = 1 troop), *Mon‐16* (*n* = 1 troop), *Mm2‐10* (*n* = 2 troops), *Mon‐38* (*n* = 1 troop), and *Ss13‐8* (*n* = 3 troops). Because the null alleles were not detected consistently across the majority of troops, data from these loci were retained in the analysis. Eleven loci showed deviations from Hardy–Weinberg equilibrium (HWE) in at least one troop, but none deviated from HWE in more than half the troops. Because departures from HWE are expected within a group‐living species that exhibits violations of assumptions underlying the Hardy–Weinberg model (e.g., small numbers of breeders within troops, mixing among troops, generational overlap), data from all loci were retained in the analysis. Using a Bonferroni‐corrected criterion for significance, we could not detect linkage disequilibrium among loci. We estimate that we sampled 80% of the individuals across 12 banded mongoose troops as determined from troop counts, representing nearly 50% of the troops estimated to be in our study focal area (*n* = 25). Sampling intensity varied according to troop size, latrine location, and visibility of feces, as well as individual mongoose latrine behaviors. We were not able to monitor all troops in the study area given limitations on staff and resources. Gaps in sampled troops exist particularly in the national park between CGL and CCH, where radio collar loss is higher and collar deployment is more challenging given dense vegetation and dangerous wildlife species that limit vehicle access and prevent following animals on foot. We use these samples and associated data to estimate local population genetic structure and individual dispersal.

Genetic diversity metrics for the 12 banded mongoose troops that we screened for microsatellite DNA variation are presented in Table 2. Observed heterozygosity, *H*_O_, tended to be somewhat smaller than expected heterozygosity, *H*_E_. Numbers of alleles per locus ranged from 2.93 in troop WA‐WP to 5.4 in troop CSL. *M*‐ratios were less than the criterion value of 0.7 (Garza & Williamson, 2001), suggesting that microsatellite alleles have been lost to recent random genetic drift. While most *F*_IS_ values were near zero, larger departures were positive, suggesting inbreeding in some troops (CSL, WA‐WP, and MOW). Private alleles were observed in nine troops, mostly at frequencies less than 0.10. Troop CGL exhibited a 141‐bp allele at locus *Mon‐38* at a frequency of 0.136.

**TABLE 2:** Genetic diversity metrics for 12 banded mongoose troops: *H*_O_ = observed heterozygosity, *H*_E_ = expected heterozygosity, *A* = mean number of alleles observed per locus, Range = mean difference in number of motif repeats between smallest and largest microsatellite alleles observed, *M*‐ratio = *A*/Range, and *F*_IS_ = within‐troop departures from Hardy–Weinberg‐expected genotype frequencies

Troop	*H_O_*	*H_E_*	*A*	Range	*M‐*ratio^a^	*F*_IS_
CGL	0.636	0.660	4.13	13.73	0.301	0.038
CCH	0.646	0.651	4.87	17.73	0.275	0.008
CSL	0.570	0.656	5.40	19.73	0.274	0.131
WDL	0.676	0.661	3.93	19.29	0.203	−0.044
FOR	0.578	0.576	3.93	16.80	0.234	−0.004
LIB	0.619	0.674	4.23	15.23	0.277	0.064
MOGO	0.665	0.651	5.00	19.47	0.257	−0.037
PLAT	0.536	0.571	4.13	16.40	0.252	0.050
WA‐WP	0.452	0.585	2.93	15.00	0.195	0.250
MOW	0.579	0.648	4.86	17.57	0.277	0.104
SEF	0.674	0.704	5.00	19.29	0.259	0.040
KUBU‐KWA	0.586	0.584	4.93	18.80	0.262	−0.004
Mean	0.601	0.635	4.45	17.42	0.256	0.050

^a^ *M*‐ratios of less than 0.7 are indicative of loss of microsatellite alleles to recent random genetic drift (Garza & Williamson, 2001).

### 3.2 Population structure and differentiation

**FIGURE 3:**
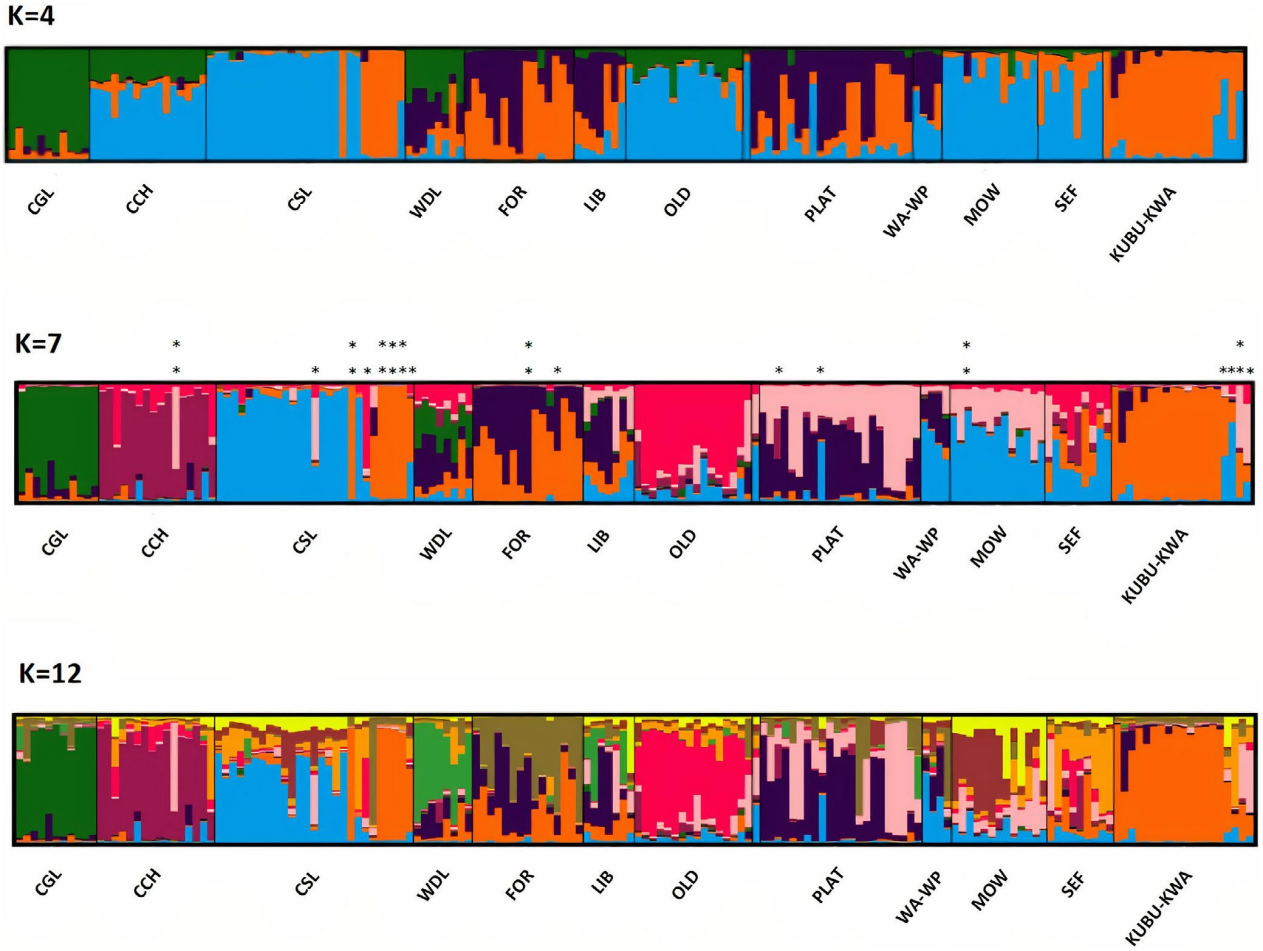
Genetic structure of banded mongoose troops in northern Botswana inferred using program STRUCTURE for *K* = 4, 7, or 12 multilocus genotypic clusters. Troops are arranged in sequential order based on geographic location from east to west. Each histogram bar shows the probability coefficients (*q*) for each individual reflecting individual assignment to seven inferred genetic clusters (*K*) using the LOCPRIOR model. Troop WA had merged with troop WP, and troop KUBU had joined with nearby troop KWA at the time of sampling. Asterisks above the bar diagram for *K* = 7 show inferred migrants (two asterisks) and offspring of migrants (one asterisk)

Using the Evanno et al. (2005) ad‐hoc ∆*K* statistic, *K* = 2 was the best‐supported number of clusters; the Evanno et al. (2005) method, however, has a known tendency to support the choice of *K* = 2 clusters (Puechmaille, 2016), and the pattern of clustering and the resulting individual assignments offered no particular insights. At *K* = 4 (Figure 3), the CGL troop at the west and the KUBU‐KWA troop at the east end of the study area present the most distinctive clusters, with little signature of mixing with others. Troop CSL also showed little signature of mixing, but most other troops, which are closely associated geographically show signatures of varying degrees of mixing. In particular, troops WDL, FOR, LIB, OLD, PLAT, and WA‐WP contained individuals that were apparent admixtures of two or more multilocus genotypic clusters. Based on the highest mean estimate of the log probability of the data (Ln*P*(*D|K*)) calculated by STRUCTURE, *K* = 7 was the most likely number of multilocus genotypic clusters. While varying levels of admixture were observed across troops, genetic signatures unique to particular troops were observed. For example, troops CGL, CCH, CSL, MOGO, and KUBU‐KWA predominantly showed individual membership within their own genetic cluster. However, there were individuals within some of these troops that exhibited high probabilities of belonging to a genetic cluster other than the troop from which they were sampled. On the other hand, certain troops—WDL, LIB, and SEF—appeared to be the result of several distinct genetic clusters having merged. The Ln*P*(*D*|*K*) criterion also showed considerable support for *K* = 12 clusters. Overall, the pattern of clusters was similar to that for *K* = 7, although certain troops (CSL, FOR, PLAT, and MOW) showed small contributions from clusters not evident at lower values of *K*. These were troops that already had shown signatures of mixing from other troops at lower values of *K*.

*F*_ST_ and *R*_ST_ metrics of genetic differentiation (Table 3) indicated that considerable genetic differentiation exists among troops in the Botswana study population. Mean *F*_ST_ and *R*_ST_ values for all pairwise comparisons between troops were 0.086 and 0.076, respectively. The mean *F*_ST_ of troops residing in the urbanized area of Kasane (CSL, WDL, FOR, LIB, MOGO, PLAT, WA‐WP, MOW, and SEF) was 0.081, which was lower than the average *F*_ST_ values for troops living in the natural landscape of the Chobe National Park (CGL and CCH, average = 0.108) and in the mixed‐use area of Kazungula (KUBU‐KWA; average = 0.086). The lowest *F*_ST_ value was found for the urban troop LIB (0.063), and the largest was found for the protected area troop CGL (0.126). The *F*_ST_ value between grouped “park” and “town” troops in our study was 0.0466, which was significant at the *p* < 0.05 level. A Mantel test for isolation‐by‐distance measured as *F*_ST_ showed a low slope (0.0018) and a poor fit of points to the regression (*R* ^2^ = 0.078). Similarly, regression of *F*_ST_/(1 − *F*_ST_) on geographic distance yielded a small regression coefficient (0.0021) and poor fit of data points to the regression (*R* ^2^ = 0.080). We concluded that isolation‐by‐distance does not apply at this spatial scale.

**TABLE 3:** Pairwise *F*_ST_ values among 12 mongoose troops. Bold font indicates values that are statistically significant (*α* = 0.05) after tests of 10,100 permutations

	CGL	CCH	CSL	WDL	FOR	LIB	MOGO	PLAT	WA‐WP	MOW	SEF	KUBU‐KWA
CGL	–											
CCH	**0.118**	–										
CSL	**0.104**	**0.066**	–									
WDL	**0.098**	**0.075**	**0.050**	–								
FOR	**0.126**	**0.132**	**0.098**	**0.075**	–							
LIB	**0.109**	**0.089**	**0.044**	0.035	**0.055**	–						
MOGO	**0.130**	**0.062**	**0.046**	**0.076**	**0.144**	**0.069**	–					
PLAT	**0.146**	**0.097**	**0.068**	**0.050**	**0.078**	0.023	**0.078**	–				
WA‐WP	**0.148**	**0.122**	0.033	**0.110**	**0.104**	**0.094**	**0.116**	**0.083**	–			
MOW	**0.167**	**0.097**	**0.032**	**0.107**	**0.177**	**0.067**	**0.046**	**0.097**	**0.100**	–		
SEF	**0.118**	**0.050**	0.015	**0.068**	**0.111**	**0.062**	**0.034**	**0.085**	**0.089**	**0.053**	–	
KUBU‐KWA	**0.120**	**0.091**	**0.062**	**0.056**	**0.085**	**0.052**	**0.100**	**0.082**	**0.130**	**0.107**	**0.064**	–

Results from the global AMOVA (Table 1) indicated that ~8% of genetic variation was among troops, ~5% among individuals within troops, and 87% within individual mongooses across troops.

### 3.3 Group relatedness

Results from analysis of relatedness (Table 4) showed considerably higher values within (mean = 0.127) than between troops (mean = −0.018, Table 4); these values, respectively, suggest some level of relatedness within troops and none between troops. Although some troops such as CSL and SEF had intragroup relatedness values relatively close to zero, which indicates little relatedness, all within‐troop values were considerably larger than pairwise comparisons among troops.

**TABLE 4:** Within‐group (along diagonal)^1^ and between‐group (off diagonal) relatedness values for 12 troops of banded mongoose

Troop	CGL	CCH	CSL	WDL	FOR	LIB	MOGO	PLAT	WA‐WP	MOW	SEF	KUBU‐KWA
CGL	0.156											
CCH	−0.155	0.109										
CSL	−0.123	−0.085	0.034									
WDL	−0.147	−0.118	−0.068	0.130								
FOR	−0.170	−0.162	−0.085	−0.137	0.129							
LIB	−0.150	−0.132	−0.045	−0.103	−0.100	0.131						
MOGO	−0.167	−0.096	−0.062	−0.118	−0.173	−0.095	0.090					
PLAT	−0.191	−0.144	−0.076	−0.101	−0.098	−0.061	−0.110	0.186				
WA‐WP	−0.167	−0.127	−0.010	−0.168	−0.134	−0.152	−0.105	−0.097	0.181			
MOW	−0.200	−0.126	−0.039	−0.156	−0.204	−0.106	−0.068	−0.125	−0.118	0.150		
SEF	−0.164	−0.093	−0.041	−0.134	−0.163	−0.117	−0.069	−0.142	−0.123	−0.097	0.050	
KUBU‐KWA	−0.164	−0.125	−0.068	−0.114	−0.107	−0.089	−0.131	−0.108	−0.149	−0.135	−0.119	0.180

^1^ All within‐group relatedness values were significantly different from zero statistically (*α* = 0.05) after 1,000 repetitions.

### 3.4 Effective population sizes

Estimates of *N*_e_ (Table 5) ranged from 3 to 29, with estimates for two troops being unbounded (i.e., “infinite”) at the upper confidence interval. Estimates of *N*_e_ from small samples from groups that include family structure might best be taken as indicators of the magnitude of *N*_e_ as opposed to precise estimates. For certain troops, such as CCH and MOGO, *N*_e_ estimates were approximately the number of individuals sampled. Although the sample size (*n* = 8) was among the lowest in the study site, the WDL troop by far had the largest effective population size (*N*_e_ = 28.2). Other troops, such as CGL and KUBU‐KWA, had effective population size estimates that were considerably lower than their sample sizes; these troops also had distinct genetic signatures within the STRUCTURE *Q* plot (Figure 3) and high pairwise differentiation values relative to other troops (*F*_ST_ = 0.125 and 0.086, respectively). These troops occurred at the edges of our study system, where isolation could have contributed to random drift and genetic differentiation.

**TABLE 5:** Sample sizes (*n*) and estimated effective population size (*N_e_* ± 95% confidence interval) for each of the 12 troops. The *N*_e_ estimated accounted for allele frequencies as low as 0.05 (*p*_crit_ = 0.05)

Troop	*n*	*N_e_ Values*	
		0.05 *p*_crit_	95% CI
CGL	11	3.0	2.3–6.1
CCH	16	16.3	10.7–27.8
CSL	27	13.1	10.4–16.8
WDL	8	28.8	7.2‐Infinite
FOR	15	10.7	6.4–19.2
LIB	7	8.5	2.7–65.3
MOGO	17	14.1	9.5–22.7
PLAT	22	7.9	5.4–11.3
WA‐WP	4	Infinite	2.9‐Infinite
MOW	13	7.5	4.2–12.5
SEF	9	Infinite	35.6‐Infinite
KUBU‐KWA	19	8.6	6.1–12.2

### 3.5 Dispersal, detection of first‐generation migrants, and infection status

Individual assignment using STRUCTURE and detection of first‐generation migrants using GeneClass2 led to the assignment of 148 individuals (88%) to the troop from which they were sampled. Five of the 12 troops in the study area included individuals that apparently originated from other troops (Table 6), with CSL contributing the most migrants (*n* = 8). There was no indication that troops with adjacent or overlapping home ranges exchanged more migrants than spatially distant troops. In fact, two troops on the eastern edge of the study area, SEF and KUBU‐KWA (Figure 1), produced the highest numbers of detectable migrants, nine and five, respectively (Table 6). The test for first‐generation migrants identified 11 individuals likely to have emigrated away from their parental troops (Table 6). Each first‐generation migrant identified using GeneClass2 corresponded with individuals identified from the STRUCTURE assignment test as having high *q* values for troops other than the one where they were sampled (Table 6, Figure 3). SEF had the largest number of first‐generation immigrants with four, while KUBU‐KWA had three. All the first‐generation migrants detected in the study assigned back to a natal troop within the urban land type (100%, *n* = 21, *p* = 0.03).

**TABLE 6:** Results from assignment and first‐generation migrant detection tests relating where individuals were sampled to where they were assigned. Superscripts (*m = i*) show how many individuals assigned to alternative troops were inferred first‐generation migrants

Troop of assignment	Troop where sampled											
	CGL	CCH	CSL	WDL	FOR	LIB	MOGO	PLAT	WA‐WP	MOW	SEF	KUBU‐KWA
CGL	**11**	–	–	–	–	–	–	–	–	–	–	–
CCH	–	**15**	–	–	–	–	–	–	–	–	–	–
CSL	–	–	**27**	–	1	–	–	1*^m^* ^=1^	–	–	–	–
WDL	–	–	–	**8**	–	–	–	–	–	–	–	–
FOR	–	–	1*^m^* ^=1^	–	**15**	–	–	1*^m^* ^=1^	––	–	–	–
LIB	–	––	–	–	–	**7**	–	–	–	–	–	1
MOGO	–	––	1	–	–	–	**17**	––	–	–	–	–
PLAT	–	–	–	–	–	–	1*^m^* ^=1^	**22**	–	–	–	–
WA‐WP	–	–	–	–	–	–	–	–	**4**	–	–	–
MOW	–	–	–	–	–	–	–	–	–	**13**	–	–
SEF	–	1*^m^* ^=1^	2	–	–	–	–	2	–	1*^m^* ^=1^	**9**	3*^m^* ^=2^
KUBU‐KWA	–	–	4*^m^* ^=2^	–	1*^m^* ^=1^	–	–	–	–	–	–	**19**

Eleven of our genotyped mongooses were confirmed on postmortem examination as being infected with *M. mungi*. Results of GeneClass2 analyses assigned eight of these individuals with high probability to the troop from which they were sampled. However, three of these infected mongooses were identified as migrants (sampled in troop CCH) with a higher assignment probability to a neighboring troop, CSL.

## 4 DISCUSSION

Among factors contributing to the dynamics of disease transmission across a landscape, host movements can play a central role by connecting individuals across the landscape and affecting pathogen transmission potential and movement (Alexander, Carlson, et al., 2018; Altizer et al., 2015; Fèvre et al., 2006; Gaidet et al., 2010; Morse, 2001). Social behaviors of a species influence how individuals disperse through a given area, especially for species that are highly territorial (Craft et al., 2011; Cross et al., 2009). Here, territorial behavior may be expected to inhibit pathogen spread if dispersing individuals are met agonistically in enemy home ranges or conversely facilitate spread if the disperser(s) successfully moves to a new habitat patch and social group. Landscape features also may affect the movement behaviors of a host. This is particularly true for wildlife species that inhabit urbanized environments where storage and disposal of human waste may affect how far individuals or groups of individuals will travel in search of resources and how they will congregate if the resource is abundant (Laver & Alexander, 2018). Data from this study suggest that land use may influence dispersal behaviors, population structure, and, ultimately, pathogen transmission potential among banded mongoose in Northern Botswana.

### 4.1 Population structure

Considerable genetic structure was evident among sampled banded mongoose troops over a scale of 20 km along the Chobe River. Among the 12 troops, highest support was provided for seven clusters of multilocus genotypes. Certain troops were apparent mixtures of individuals from two or more genetically distinct source groups. Such troops could be the result of individuals or groups from different troops either voluntarily leaving or being forcibly evicted (Cant et al., 2001, 2010) and subsequently coming together to form a new troop in an available territory (group fusion). Our STRUCTURE results suggest mixing of individuals originating from multiple troops in KUBU‐KWA (a troop fused between KUBU and KWA), MOGO, and PLAT within‐troop CSL (Figure 3). The fused troop KUBU‐KWA also appeared to have additional individuals from outside of the troop. Several troops appear to have individuals that are admixed, with ancestry suggesting origins from multiple troops. For example, individuals from troop CGL may have joined individuals from troop LIB or other troops to interbreed and establish troop WDL, which shows ancestry from multiple source troops. The interpretations of mixing among troops were evident at other levels of *K* as well. Average *F*_ST_ values for troop and natural parks were significantly different (*p* < 0.05) with urban troops having lower values. These data suggest that troops living in the urban areas had higher levels of gene flow. We note that troops within urban environments were more genetically similar than troops in mixed‐use and protected land areas, suggesting higher levels of individual dispersal and gene flow in anthropogenic landscapes, habitat types that were not within the Ugandan mongoose study in Queen Elizabeth National Park (Nichols et al., 2012).

Anthropogenic environments may facilitate intertroop movement. Troops living in urbanized areas with abundant food resources (i.e., human garbage, with larger and more calorie‐dense foods than insects) may be more receptive to immigrants than troops living in natural ecosystems where denning and natural food resources (restricted to insects and small vertebrates) are more limiting. The abundant denning and food resources that are often available in urban landscapes have been shown previously to facilitate elevated levels of congregation in this species and others that are able to adapt and thrive in anthropogenic habitats (Bateman & Fleming, 2012; Bradley & Altizer, 2007; DeStefano & DeGraaf, 2003; Hassell et al., 2017). These findings are consistent with previous results from our study system demonstrating the importance of the urban landscape on species behavior and pathogen transmission potential. Mongoose troops living in these anthropogenic landscapes had smaller home ranges in the dry season and concentrated space use around buildings and human refuse (Laver & Alexander, 2018). Urban areas also appeared to relax territorial behaviors, with den sharing occurring among troops living in these land areas (Nichols & Alexander, 2019).

### 4.2 Dispersal

We found high levels of dispersal of individuals into established troops (12.5% of genotyped individuals), in contrast to the Uganda population where dispersal of this nature was infrequent to absent among study troops (Nichols et al., 2012). Overall, 21 apparent migrants or dispersing mongooses were detected among the 168 multilocus genotypes analyzed using GeneClass2 (Table 6). From this pool of individuals, 11 were identified as first‐generation migrants, themselves having dispersed from their inferred natal troop. There was no indication, however, that troops with adjacent or overlapping home ranges exchanged more migrants than spatially distant troops; the correlation of pairwise genetic differentiation among given troops and geographic distance between them yielded an *R* ^2^ value of just 0.159.

Our results from GeneClass2 analysis assigned 73% (*n* = 11) of *M. mungi‐*infected individuals to the troop from which they were sampled. This appears to be consistent with previous observational studies (Fairbanks et al., 2014) identifying that clinically infected banded mongooses tended not to disperse as frequently as putatively healthy individuals. In our dataset, there were three individuals originating from troop CCH that had a higher assignment probability to neighboring troop CSL. These mongooses could have been infected after dispersal or moved, while in the latent stages of infection, a period of time that is still uncertain for this pathogen. Given TB latency and the low numbers of infected individuals evaluated in this study, our data do not provide a strong test of the effect of TB infection upon dispersal behavior.

Anthropogenic habitats also may influence gene flow by nonconventional modes of dispersal. Human‐mediated movement of wildlife can have important impacts on gene flow (Banks et al., 2015; Capinha et al., 2015; Waterkeyn et al., 2010) influencing, in turn, the potential for pathogen movement. In our context, banded mongooses can be moved incidental to human activities, crossing distances that normal ecological conditions likely would not allow. For example, genotyping and individual assignment test results obtained for an orphaned mongoose pup found by a Kazungula fisherman showed that it was most likely a member of MOGO troop near Kasane. The 8‐km journey was an unrealistic geographic distance for pup movement outside of the natal area, and human‐mediated transport appears likely.

With troops living in close proximity, some with overlapping home ranges (Laver & Alexander, 2018; Nichols & Alexander, 2019), a mean genetic differentiation metric *F*_ST_ of 0.086 across all land uses was relatively high, especially given evidence of intertroop movements and the limited size of our study area. Movement between banded mongoose troops in Uganda was more limited, but mating between groups was reasonably common, with around 20% of pups being the product of extra‐group mating. Gene flow between groups therefore does occur, but without the levels of migration observed in Botswana; while several of the groups in Uganda were in an area of human activity, with access to refuse and anthropogenic den sites, immigration into these groups was rare, so there may be other factors at play. In the future, it would be important to sample more troops within the protected area in Botswana to further advance our understanding of the influence of urbanization on behavior and troop dynamics in this species.

As noted above, the *F*_ST_ between grouped “park” and “town” troops in Botswana was 0.047. In comparison, *F*_ST_ values for mongoose troops in Uganda (average *F*_ST_ = 0.129, Nichols et al., 2012) were higher than those in this study; the Ugandan mongoose troops, as noted previously, occurred in protected areas, and individual movement between troops was exceptionally limited. Troops in our study area living in the town Kasane and the mixed‐use area of Kazungula had lower mean *F*_ST_ values (mean *F*_ST_ = 0.081 and 0.086, respectively) than troops living in natural environments (mean *F*_ST_ = 0.108), the latter value comparable to that identified for the study troops in Uganda.

Previous studies of banded mongoose behavior (Gusset, 2007; Nichols et al., 2012) have shown that individuals are typically philopatric and often mate within their natal group. Findings from our troop relatedness analysis suggest that banded mongoose practice similar behaviors in our study site. Although detection of mixture and admixture (Figure 2) and dispersal events (Table 6) suggests that there are some familial relationships among troops, relatedness values within troops were generally higher than between‐troop values (Table 4, Figure 3). As a result of intragroup breeding, smaller effective population sizes would be expected for long‐established troops in the study system, as was seen for troops CGL (*N*_e_ = 3.0; troop size = 11) and KUBU‐KWA (*N*_e_ = 8.6; troop size = 19); troop sizes reported here are the numbers of individuals sampled; for 8 out of the 12 troops, we sampled two or more different mornings to ensure that we represented as many individuals as possible, and we chose to use fecal samples from the days on which we collected the greatest number of scats from a troop. Genotype frequencies in these well‐established troops may be more strongly influenced by random genetic drift (mean *F*_ST_ for CGL = 0.125, for KUBU‐KWA mean *F*_ST_ = 0.086) than by recent gene flow, as multiple generations of individuals have stayed within the natal troop and the inflow of novel genotypes decreases. The opposite dynamic may be inferred for newer troops, such as WDL and LIB, with few members (8 and 7, respectively), but substantially higher estimates of *N*_e_ (28.8 and 8.5, respectively). These smaller troops with higher genotypic variability and admixture likely formed recently through the joining of individuals or cohorts from genetically distinct natal troops, a dynamic suggested by Nichols et al. (2012), possibly explaining their large effective population sizes. As a new troop breeds within itself over time, drift would push *N*_e_ to decline as generations of newly recruited pups remain philopatric. Troops CGL and KUBU‐KWA are also the most distant from other troops studied and hence less linked to others by gene flow, which would exacerbate random genetic drift. The longevity of the respective troops in our system differs. The earliest record for CGL is 2000, around the time the study of the banded mongoose system started at the site. The earliest record for KUBU‐KWA was 2012, and 2017 for WDL and LIB. Ongoing analysis of microsatellite markers amplified from DNA derived from mongoose feces and tissues collected across a decadal time‐step (2008, *n* = 51) and 2017, (*n* = 86) showed that the genetic structuring of troops in our study area was largely consistent over time, although more mixture was detected in 2017. Our field data suggest that troop membership in our study area turns over within a few years, although we do not have datasets as detailed as those for banded mongoose in other systems. In Uganda, the average age of first conception is at about one year of age (Gilchrist et al., 2004), and in Kenya about two (Waser et al., 1995). Mean average annual adult survivorship is 0.67 in Kenya (Waser et al., 1995) and 0.78 to 0.86 in Uganda (Cant, 1998; Otali & Gilchrist, 2004). Among individuals surviving to one year in Uganda, males lived an average of 42 months and females 38 months (Cant et al., 2016).

Based on the results of parentage and relatedness analysis, Nichols et al. (2014) found that female banded mongoose in Uganda breed with close relatives, suggesting the cost of inbreeding avoidance may outweigh the benefits under certain circumstances. However, where extra‐group matings did occur, pups in the Uganda population were genetically more heterozygous, heavier, and had a greater likelihood of survival to independence (Nichols et al., 2015), benefits that would appear to offset risks. In Botswana, extra‐group matings have also been observed during agonistic troop encounters. Additionally, we have observed nocturnal excursions by lone mongoose and investigation of occupied den sites in other mongoose territories, the purpose of which is uncertain (Nichols & Alexander, 2018). These behaviors suggest that gene flow may also arise from other types of intra‐troop contacts beyond dispersal events where breeding opportunities arise and are exploited. Extra‐group breeding behaviors are expected to be influenced by adaptive fitness advantages that would be predicted to shift in respect of troop history and heterozygosity. For example, in early establishment with higher levels of troop heterozygosity (e.g., troop fusion), extra‐troop breeding would presumably be less adaptive and less common as compared to older troops where extended maintenance of the group as a semi‐closed social unit would be predicted to reduce heterozygosity, increasing the fitness benefits of extra‐group matings. In our study site, land use would appear to have an important influence on these dynamics. In this system, disease also has a potential to influence potential fitness advantages of extra‐troop breeding with agonistic interactions increasing the potential risk of injury and *M. mungi* disease transmission (Alexander, Laver, et al., 2018; Flint et al., 2016).

### 4.3 Urban landscapes, species behavior, and infectious disease transmission

Considerable attention has been directed at the manner in which landscape transformation and urbanization contribute to habitat loss, movement, and fragmentation, in particular, how these processes can decrease landscape connectivity and species movement (Bradley & Altizer, 2007; Liu et al., 2009; Lowry et al., 2013; Noël et al., 2007; Tremblay & St Clair, 2009). Our data suggest that urban landscapes may increase population connectivity for banded mongoose in northern Botswana, at least within this land type. Heightened connectivity may, in turn, increase the potential for pathogen transmission in socially structured populations that are able to adapt to anthropogenic landscapes, an inference central to disease modeling efforts and intervention design. However, infection status itself may influence individual dispersal behavior, further complicating our ability to predict landscape–host–pathogen interactions and disease spread. As urban landscapes across the globe grow, there is increasing pressure to understand how these growing landscapes influence disease transmission and persistence, potentially escalating the risk of disease transmission in both human and animals.

## CONFLICT OF INTEREST

The authors state that they have no conflict of interest.

## AUTHOR CONTRIBUTION

**Kelton Verble:** Conceptualization (supporting); Data curation (lead); Formal analysis (lead); Investigation (lead); Methodology (equal); Validation (lead); Visualization (lead); Writing‐original draft (equal); Writing‐review & editing (equal). **Eric Hallerman:** Conceptualization (supporting); Data curation (supporting); Formal analysis (supporting); Investigation (supporting); Methodology (equal); Resources (supporting); Supervision (equal); Validation (supporting); Visualization (supporting); Writing‐original draft (equal); Writing‐review & editing (equal). **Kathleen A Alexander:** Conceptualization (lead); Data curation (supporting); Formal analysis (supporting); Funding acquisition (lead); Investigation (supporting); Methodology (supporting); Project administration (lead); Resources (lead); Supervision (equal); Validation (supporting); Visualization (supporting); Writing‐original draft (equal); Writing‐review & editing (supporting).

## Data Availability

The data that support the findings of this study are openly available in University Libraries, Virginia Tech at https://doi.org/10.7294/29KX‐E267.
